# Factors affecting the motivation of community health workers: Perspectives from Accredited Social Health Activists (ASHA) in Uttar Pradesh, India

**DOI:** 10.1371/journal.pone.0341811

**Published:** 2026-02-02

**Authors:** Dorinda ’t Hart, Jaya Menon, Dani J. Barrington, Arshe Alam, John Hembling, Deepti Pant, Rahul Dutta, Timothy Roberton

**Affiliations:** 1 School of Population and Global Health, University of Western Australia, Perth, Western Australia, Australia; 2 Catholic Relief Services, Lucknow, Uttar Pradesh, India; 3 Catholic Relief Services, Baltimore, Maryland, United States of America; University College of Medical Sciences, INDIA

## Abstract

**Background:**

Accredited Social Health Activists (ASHAs) are critical to extending health services to rural and underserved populations in India. Understanding what motivates these community health workers is essential for health organizations seeking to optimize their performance and retention.

**Objective:**

To explore factors affecting the motivation of ASHAs in Uttar Pradesh, India, from the perspectives of the ASHAs themselves.

**Methods:**

Qualitative in-depth interviews were conducted with 40 ASHAs across ten districts in Uttar Pradesh between September 6–24, 2023. ASHAs were selected based on the performance of their supervising ASHA Sanginis. Interviews were transcribed, translated from Hindi to English, and analyzed using thematic analysis in NVivo 12.

**Results:**

Two categories of motivating factors emerged. Personal and community factors included self-efficacy from acquiring new knowledge, satisfaction from contributing to community health, increased autonomy and empowerment, and gaining respect within their communities. Organizational factors included training and skills development, supportive supervision from ASHA Sanginis, and financial incentives. While ASHAs remained positive about their work despite challenges, delayed payments and inadequate training were identified as key demotivating factors.

**Conclusions:**

Health organizations can leverage organizational factors – adequate and timely training, supportive supervision, well-stocked drug kits, and timely remuneration – to support ASHAs’ intrinsic motivation. Failure to address these factors risks demotivation, reduced performance, and poorer health outcomes for the communities ASHAs serve.

## Background

The Accredited Social Health Activist (ASHA) program was established in 2005 by the Indian Ministry of Health and Family Welfare Department, to improve reproductive, maternal, and child health across the country [1], particularly in hard-to-reach areas. ASHAs (the Indian equivalent of community health workers in many low- and middle-income countries), live in, and are selected by their community at a rate of ~1 ASHA per 1000 population [2]. They provide a vital link between health services and the community, particularly those in rural and under-served areas [3,4], providing a promising strategy to improve maternal and child health outcomes.

The women chosen for ASHA roles often have limited education and often work in isolated locations with limited support [5]. After a series of training modules, an ASHA is equipped to provide medical information to the women in their designated area – an expected population of 700 in tribal areas and 1000 in rural villages [6]. Her main task is to visit the women’s homes to counsel them on sexual and reproductive health, identify pregnancies and encourage all women and particularly those with High-Risk Pregnancies (HRP) to have an institutional birth, do health checks on newborns and advise mothers on neonatal care [6,7]. ASHAs do not receive a salary, but do receive incentive payments for completing certain tasks [8].

Because of the nature of the work – the unsalaried position, the low level of training offered and the vital link between rural communities and health services – it is important to consider what factors maintain and support an ASHA’s motivation for the sustainability of such programs [8]. Previous studies have identified motivation as a key driver for retention of health workers, with the expectation that motivated community health workers will perform better [3,9,10], maximizing their impact on goals such as improving maternal and child health [11,12]. Conversely, low motivation has been shown to negatively impact ASHA performance, leading to less frequent beneficiary visits and thus worse health outcomes [11,13]. This means that the ongoing motivation of ASHAs is critical to health service delivery, directly impacting the quality of work at an individual and organizational level [14,15].

Previous studies examining motivational factors for health workers have defined motivation as the “individual’s degree of willingness to exert and maintain an effort towards organizational goals” [9,16], consisting of both personal and community factors, and organizational factors [3,16]. Prior studies have shown that by creating an environment in which the ASHA receives ongoing training, is adequately equipped, has strong supervision support, and adequate financial incentives, the organization can positively impact a health worker’s ongoing motivation [5,6,9].These motivational elements increase the ASHA’s capacity for performing her task well, which contributes to greater reach of health services and improved health outcomes [3,9].

The data for this paper came from a larger, co-designed study which used qualitative and quantitative tools to evaluate the effectiveness of a technological initiative to aid ASHA supervision in Uttar Pradesh, India. Participants included ASHAs, ASHA Sanginis (supervisors of ASHAs) and Health Officials [17]. During the qualitative interviews, ASHAs had the opportunity to speak about their general experiences of being an ASHA (See Supplementary Material A). Some shared aspects that were both enjoyable and challenging. These comments were also analyzed thematically in Nvivo 12. This paper explores the comments that ASHAs made regarding motivation, highlighting the factors mentioned by ASHAs that relate to their experience both personally and in their community, and the organizational factors that support or demotivate them in their work.

## Methods

### Study context

The study was led by Catholic Relief Services (CRS) in India in conjunction with three researchers from the University of Western Australia (UWA). CRS outsourced the data collection to a local Indian firm, Catalyst Foundation, with significant experience of quantitative and qualitative research methods. The UWA team designed project-specific training sessions for the Catalyst Foundation leadership team and CRS to run with the data collectors. The local team (CRS and Catalyst Foundation) kept weekly contact with the UWA researchers via phone meetings and emails, as well as sharing the qualitative data as it was collected, transcribed and translated, for continuous feedback and improvement of the process. Here we discuss the data collected and analyzed that is relevant to ASHA motivation.

### Sampling and recruitment

As part of the larger, co-designed project, four ASHA Sanginis were purposefully sampled from each of the ten districts under evaluation. Based on task completion in the (phone/tablet) application being evaluated, two strong performing ASHA Sanginis (those completing 100% of mandatory monthly ASHA supervision visits for the prior three months, with at least 75% of their ASHAs functional on at least 60% of tasks) and two ASHA Sanginis in need of further support (those with less than 60% of mandated monthly ASHA supervisory visits) were randomly selected. For logistical reasons, we sampled ASHAs based on the sampled Sanginis. In each district, we randomly selected two of the four sampled Sanginis, and for each of these, we randomly sampled two ASHAs from the Sangini’s list of ASHAs (n = 40). Data collectors met with the 40 ASHAs outside of their normal working hours, and the ASHAs were not given any financial incentive to participate. Saturation was achieved during these 40 interviews [17].

### Study methods

CRS data collectors, who were fully trained in the study’s protocols, conducted the in-depth interviews, which typically lasted 30–60 minutes. The interviews focused primarily on the main objectives of the larger study, using an interview guide prepared together with UWA researchers (See Supplementary Material A). As is often the case with in-depth interviews, ASHAs also had the opportunity to comment on what made them feel good about their work and what factors enabled them to best perform their duties. It is these comments that were further analyzed and are presented in this paper.

The interviews were recorded, transcribed verbatim and then translated from Hindi into English. Transcripts were then de-identified and sent to the first author, who imported them into Nvivo 12 for analysis. Using the link identified in the literature between motivation and performance, and the important role of supervision to support the ASHA, the interviews were analyzed using thematic analysis, creating *in vivo* themes that spoke to their motivation and well-being, as well as their experiences of supervision and working within the community.

### Ethics

Ethical approval was received from the Catalyst Foundation Institutional Ethics Committee in India (the committee does not provide a study number). All participants provided informed, written consent before interviews took place.

## Results

In this section we highlight the factors that ASHAs refer to, either explicitly or implicitly, as motivating or demotivating. We first present the results on factors that appear to be more personal in nature, relating to the individual ASHA or their relationship with community members. We then present findings on factors that relate more to the implementation of the ASHA program, and that sit within the control of the National Health Mission (NHM). (See Table 1). All quotes are presented using participants’ role and area location (e.g., ASHA #, Sitapur), not personal identifiers.

**Table 1:** Themes, Sub-themes and definitions.

Theme	Sub-theme	Description
**Personal & Community Factors**	Self-efficacy	Increased confidence from learning new skills; feeling capable of performing ASHA duties
	Contribution to community	Satisfaction from helping women and families; facilitating good health outcomes
	Autonomy & empowerment	Independence to leave house and interact with community; financial security; reduced family dependence
	Respect	Gaining social recognition and status within the community over time
**Organizational Factors**	Training & skills development	Ongoing training builds knowledge and capacity; lack of training undermines trust and performance
	Being adequately equipped	Drug kits with supplies and medicines. Without supplies, ASHAs cannot perform tasks effectively
	Supportive supervision	Regular visits from ASHA Sangini; encouragement and correction; reinforcing ASHA credibility with beneficiaries
	Financial incentives	Task-based payments provide income and security; delayed or inadequate payments are demotivating

### Personal and community factors

In this section, we present findings that are often personal in nature and indicate the ASHAs’ strong commitment to their work. These factors were generally captured in comments about learning new things to ‘help women’ in their community. Many ASHAs said that seeing women make greater use of health services was satisfying and made them feel good about their work. In this section, we also present findings that relate to the ASHA’s relationship with their community. Working within their own community meant they were able to use their knowledge to directly improve community access to health services but could also create certain challenges, such as having to earn the community’s respect.

#### Self-efficacy.

Some ASHAs reported feeling a boost in confidence due to learning new things. This knowledge had a two-fold effect in that it increased the ASHA’s sense of self and improved her skills to perform her task.

I used to cover my face, but now I have the confidence to perform as an ASHA while having my face exposed. I have time now to leave my place. I enjoy getting to interact with new individuals and having conversations with them (ASHA 1, Sitapur).

I have also gained knowledge gradually on different aspects of health care. I made my identity and gained the respect of the people (ASHA 3, Lakhimpur Kheeri).

#### Contribution to community.

Some ASHAs reported that they enjoyed interacting with women and families within their community. They reported feeling satisfied when they were able to facilitate good outcomes for the beneficiaries and their families.

I enjoy taking care of pregnant women, arranging vaccinations, helping people, going to their homes, asking about their problems, and finding solutions (ASHA 1, Kannauj).

Going to people’s homes, talking to them, and helping them as much as possible is what I enjoy the most (ASHA 1, Bareilly).

I make an effort to serve beneficiaries, meaning we provide health care to them so that they can have good health. I find satisfaction in this, and I am happy that we can help people and make them happy (ASHA 1, Ayodhya).

#### Autonomy and empowerment.

Some ASHAs reported that their work gave them a reason to leave the house and interact with the women of their village. They learned to be more independent and felt more secure because of the new skills and relationships that they had acquired and because of their increased financial security.

I feel good about it. If I hadn’t become an ASHA worker, I wouldn’t have been allowed to leave my house. Working as an ASHA worker, I can connect with people, provide services, and even earn some respect in the village. It also adds to the security (ASHA 2, Mizapur).

Since I became an ASHA, I have become more preoccupied with my work and pay less attention to the little things that go on at home. My body language has changed and I am more confident in speaking up in front of a group of people. Prior to this, I was reliant on my husband or my in-laws for financial matters. My relatives and neighbours now appreciate me, and there are less arguments within the family. My mother-in-law has begun assisting with housework (ASHA 2, Lakhimpur Kheeri).

#### Respect.

Some ASHAs noted that while they were not always respected initially, they found that over time, they had gained respect in their role. With the support of the ASHA Sanginis, they were able to demonstrate to the people in their district that they did have the knowledge and capacity to fulfill their role, especially when good outcomes were achieved.

Now things are executed in a better way because the villagers pay greater attention to the Sangini and me when we go together. Otherwise, the old women don’t pay much heed to the advice that I give to their daughters-in-law and start comparing it to their times. People have now become more aware of the health conditions (ASHA 3, Lakhimpur Kheeri).

It feels good to go to every house in the village, talk to everyone, and provide services to the beneficiaries. It feels good when I get respect in the village. People offer me food and water. It feels good. No one knows what we do when I stay at home. But when I go out as an ASHA worker and attend meetings, everyone knows me and understands my work (ASHA 1, Mirzapur).

Since I started working as an ASHA worker in 2007, there have been many changes. People in the village have started respecting me, they listen to me, and if there are any issues, they inform me. It has been a positive experience (ASHA 2, Bareilly).

### Organizational factors

ASHAs also made comments about the structural aspects of their work which either motivated them or de-motivated them. These are external factors that the ASHA may not be able to control as they usually sit within the realm and control of the NHM. For example, good use of resources such as training and supervisory practices enabled the ASHAs to work confidently and enjoy their role, while the absence or need for improvements in these areas tended to create challenges for the ASHAs.

#### Training and skills development.

National guidelines recommend that ASHAs receive 23 days of training in their first year and 12 days of training every year after that [18]. The ASHAs reported positively that they felt that they had learned new things through their work.

Sister (ASHA Sangini) was doing well; we take her help. We receive training from Sister, and she guides us in everything (ASHA 1, Ayodhya).

The experience has been good. Initially, I didn’t know much about high-risk pregnancies or other things, and I didn’t have much experience either. But gradually, I learned and my experience increased (ASHA 3, Bareilly).

At the same time, some of the ASHAs complained that they had to wait a long time for a short training session, which seemed to de-value them.

We haven’t received any specific training in these five years as an ASHA worker. We keep requesting the higher authorities to provide training, but they keep saying that there’s no budget for it (ASHA 1, Gonda).

#### Being adequately equipped.

Similarly, a common problem that surfaced in the ASHA interviews was that their drug kits, containing basic equipment and medicines, were not (regularly) replenished, which also hindered the ASHAs in their work. Some ASHAs spoke about medicines and the necessity of checking the expiry dates before administration.

I have condoms and iron tablets. When we go to someone’s house, Sister checks the expiry date first and then gives the medicine. We don’t give it without checking (ASHA 2, Prayagraj).

Others identified that they lacked medicine supply which hindered them in their work.

The problem is, Didi (‘big sister’, a term of respect for an older woman), I don’t have all the supplies, and I haven’t received complete training, so people don’t trust me either (ASHA 1, Kanpur Nagar).

#### Supportive supervision.

Commonly, when the ASHA Sangini arrived for a supervisory visit, she would first enquire about the ASHA’s wellbeing. Sometimes they would drink tea together before commencing with the work, checking the ASHA diary and visiting the homes of the beneficiaries. This seemed to foster a good relationship between the ASHA Sangini and the ASHA worker, so that most ASHAs reported feeling supported in their role. In this relationship, the ASHA Sangini was able to offer correction and encouragement.

The Sangini asks about me and my kids. Then she checks my diary and if she finds any missing information/work, she nicely reminds me that I need to finish it before her next visit. Then she asks me to accompany her on the visit to the houses of the HRPs (high risk pregnancies) and underweight children… She is very supportive and explains everything very politely. I do not feel that she is my supervisor, rather, I feel like she is a family member (AHSA 1, Lakhimpur Kheeri).

Sometimes, the Sangini may scold us, and it doesn’t feel good when someone scolds us. But if it’s a mistake, we’d rather they explain it to us so that we understand. It can be both hurtful and helpful (ASHA 2, Gonda).

Some ASHAs reported feeling a lack of respect from community members, particularly at the beginning. Overall, the ASHAs felt that their Sanginis provided good support during a supervisory visit from the ASHA Sangini, who would visit the beneficiary and reaffirm the advice that the ASHA had given. Over time, and because of the involvement of the Sangini, the beneficiaries came to trust the role of the ASHA within their community and to accept visits from the ASHAs.

In the village, since I live here, people don’t take my words too seriously. But when Sangini Didi comes, they perceive her as an authority figure, and they tend to listen to her more. If she suggests something important or refers someone, they take it seriously (ASHA 1, Mirzapur).

Even though I try to explain, some people don’t listen, so Sangini Didi visits them as well and explains things thoroughly. There are two or three people in our village who have also started listening to Sangini Didi (ASHA 1, Kanpur Nagar).

#### Financial incentives.

While the ASHA role is an unsalaried position, the ASHAs receive payments for set tasks [3,19]. The benefits to the ASHAs from receiving an income included a sense of financial security and independence.

I made my identity and gained the respect of the people. I am now financially secure, more confident, and can go to any place independently without any help. (ASHA 3, Lakhimpur Kheeri).

However, the issue of inadequate pay or tardiness in receiving payments was frequently mentioned at all levels – by the ASHAs, the ASHA Sanginis and the Health officials. The threat of incentive deductions was also used to motivate the ASHAs to complete their tasks. Further, if a pregnant woman goes to a private hospital at the time of delivery, her ASHA does not get paid for her service throughout the pregnancy.

Ma’am, we work very hard, but we don’t get much money, and we don’t get paid on time (ASHA 1, Gonda).

I just want to say that if we don’t get the payment on time after finishing our work, then it really upsets my mind and demotivates me. We should get the money that we deserve according to our work (ASHA 1, Budaun).

## Discussion

The findings from this study confirm existing knowledge regarding ASHAs and what motivates them personally: a desire to help women in their homes and a desire to see improved health outcomes [9]. Further benefits can be seen in the ASHA’s relationship and standing within their community. Many of the ASHAs felt an increased sense of autonomy and confidence [8]. Through the execution of their task, the ASHAs generally became more confident in leaving their house, moving about within the community, and conducting home visits to participants in national and state-level public health programs. These benefits concur with previous research that ASHAs benefit from the social recognition and enhanced social status that they receive from their role within the community [8–10]. While these personal and community factors may appear to be outside of the organization’s control, further research could investigate the role of the recruitment process in producing ASHAs who are motivated and in good standing within their community.

Although ASHAs are considered untrained, they are required to do 23 days training in their first year as an ASHA, followed by 12 days training every subsequent year [18]. This ongoing training and refresher training is part of the organizational factors that can be leveraged by the organization to support the ASHAs’ intrinsic motivations. Likewise, ASHA Sangini visits can be leveraged to support and encourage the ASHAs. According to previous studies, close and supportive relationships with supervisors was a motivating factor for many community health workers [20]. Some Sanginis in this study indicated they have changed their field visits to incorporate more hands-on demonstrations to further train ASHAs (authors, forthcoming).

However, research suggests that frustration or discouragement tends to be due to organizational factors such as payments rather than the job itself [14,19]. ASHA workers only receive incentive payments for performing certain tasks [1,6], such as beneficiary visits, successfully organizing an institutional delivery and maintaining HRP lists, due date lists and newborn lists. This situation was confirmed by the qualitative interviews with ASHAs. Despite sometimes being faced with challenges such as vaccination refusal, or institutional delivery refusal, the ASHAs remained positive and satisfied in their work. However, when payments were delayed or deductions were made due to unsatisfactory reporting, the ASHAs found these kinds of practices were demotivating. This is consistent with regular reporting in the literature of community health workers’ dissatisfaction with pay schemes [1,3,6,7,9,10,21].

Equipping ASHAs to fulfill their task by providing adequate, ongoing and timely training [22], supportive and regular supervision [20,21], drug kits that are continuously supplied [23], and adequate and timely remuneration [21] are factors that health services organizations can leverage to support the ASHAs sense of satisfaction through her intrinsic motivation.motivation. Conversely, failing to support the ASHAs in this way could lead to frustration and demotivation for the AHSAs. These demotivational outcomes may lead to reduced ASHA performance, and in turn, to less desirable health outcomes for the community. Because of the important role that ASHAs play in extending health services to rural and/or under-served populations, it is critical that health organizations are aware of the organizational factors they can leverage to ensure ASHAs are able to perform their duties well and contribute to improved maternal and child health outcomes in rural India.

## Conclusion

As ASHAs play an important role in extending health services to rural and/or under-served populations, it is critical that health organizations are aware of the organizational factors that they can leverage to ensure ASHAs are able to perform their duties well. Our study highlights that while personal and community factors also impact ASHA motivation and satisfaction, health organizations need to ensure that ASHAs are equipped to fulfill their task by providing adequate, ongoing and timely training, supportive and regular supervision, drug kits that are continuously supplied, and adequate and timely remuneration. Failing to support them in these areas could lead to frustration and demotivation for the AHSAs which could lead to poorer health services to the rural and/or under-served populations.

## Supporting information

S1 Table Themes, Sub-themes and definitions. (PDF)

S2 File Interview Guide. (PDF)

S3 File COREQ guidelines. (PDF)

## Abbreviations

ASHA: Accredited Social Health Activist
ASHA Sangini: Accredited Social Health Activist supervisor
HRP: high risk pregnancy
CRS: Catholic Relief Services
UWA: University of Western Australia
NHM: National Health Mission.
