# Supplementary material for: Factors affecting the motivation of community health workers: Perspectives from Accredited Social Health Activists (ASHA) in Uttar Pradesh, India
Source: PLoS One. 2026 Feb 2;21(2):e0341811. doi: 10.1371/journal.pone.0341811 (PMC12863482; doi:10.1371/journal.pone.0341811)
Supplement: S1 Table — (PDF) [file pone.0341811.s001.pdf]

Table 1. Themes, Sub-themes and descriptions

Theme	Sub-theme	Description
**Personal & Community Factors**	Self-efficacy	Increased confidence from learning new skills; feeling capable of performing ASHA duties
	Contribution to community	Satisfaction from helping women and families; facilitating good health outcomes
	Autonomy & empowerment	Independence to leave house and interact with community; financial security; reduced family dependence
	Respect	Gaining social recognition and status within the community over time
**Organizational Factors**	Training & skills development	Ongoing training builds knowledge and capacity; lack of training undermines trust and performance
	Supportive supervision	Regular visits from ASHA Sangini; encouragement and correction; reinforcing ASHA credibility with beneficiaries
	Being adequately equipped	Drug kits with supplies and medicines.Without supplies, ASHAs cannot perform tasks effectively
	Financial incentives	Task-based payments provide income and security; delayed or inadequate payments are demotivating
