# Supplementary material for: Factors affecting the motivation of community health workers: Perspectives from Accredited Social Health Activists (ASHA) in Uttar Pradesh, India
Source: PLoS One. 2026 Feb 2;21(2):e0341811. doi: 10.1371/journal.pone.0341811 (PMC12863482; doi:10.1371/journal.pone.0341811)
Supplement: S2 File — (PDF) [file pone.0341811.s002.pdf]

Qualitative Interview Guide – ASHA

Project Title: Impact of the ASHA Sangini Scaleup Project on frontline worker performance and key health indicators in Uttar Pradesh

Purpose, activities, and risks:

My name is ………………………………… I am doing a survey on behalf of Catholic Relief Services, which is trying to understand the impact of a project that was implemented by the National Health Mission (NHM), Uttar Pradesh, and CRS. The project involved the development of an "app" to help ASHA Sanginis provide structured supervision to ASHAs. This survey is part of a study to understand how the app affected the performance of frontline workers and data use for decision-making in Uttar Pradesh.

The purpose of this interview is to understand your overall experiences of the supportive supervision visits you received from your ASHA Sangini. This information is very important. It will help our future work in the area of supportive supervision and the use of technology to improve the performance of frontline workers, as well as improve key maternal and child indicators.

Participation and Withdrawal

Your participation in this interview will take less than an hour. Your participation is purely voluntary, and it is important that you answer each question truthfully based on your experiences. You may decide not to participate, or you may leave this interview at any time. Your decision will not result in any penalty or loss of benefits as an ASHA. We hope that you will participate in the discussion since your views are important. If you feel that you are not comfortable answering a question, please feel free to share that with us and we can move to another question. If you do not understand anything that has been explained so far or have any additional questions related to this interview, please feel free to ask them now. You can also ask the interviewer any questions you have whilst you are participating.

Compensation

For your participation in this interview, you will not receive any compensation. If you choose not to participate, it will not affect your work as an ASHA.

Privacy/Confidentiality/Data Security

Your responses to survey questions will be kept completely confidential. Only you and I know what we are discussing. Other researchers will read your responses, but they will not know that you are the person who shared those views: at no time will your identity be revealed to anyone else. The information will be used for purposes of the study only. The interview will be conducted on mobile/tablet.

- Copies of your responses will be translated into English and shared with our research partners at The University of Western Australia for analysis via *email/Teams*. Your name will not be associated with these documents in any way.
- Your personal identifiers will be stored on password and virus-protected laptops.
- All direct and indirect identifiers or codes related with your identity will be removed from the information collected during the interview. Your consent and interview data will not be merged.
- Information collected in physical form during the study (e.g., consent forms) will be safely stored in locked cabinets and no none except the local research team will have access.
- Your data will be destroyed 3 years after the research has been completed.

Audio Recording-

During the interview, audio recording devices will be used. This is required for recording the discussion, which will then be translated into English and analyzed so that we can best understand your experiences and write reports. During the interview, if at any time you do not want a certain part of the discussion recorded, please let me know and we can stop the recording. The recording will be kept in a safe drive with the research agency for 3 years once the study report is finalized. All the audio recordings will be destroyed after 3 years.
Consent/Agreement

"The nature and purpose of this study have been sufficiently explained and I agree to participate in this study. I understand that I am free to withdraw at any time without incurring any penalty."
Signature: ____

Name (printed): ____

Signature of witness: ____

Name of witness (printed): ____
Date: ______ /_______ /_______

In case you have any questions or clarifications, you may contact Mr. Arshe Alam, Senior Program Officer, Catholic Relief Services on +91 9415037874.

Note: *Both the researcher and the participant should receive a signed copy of the consent form.*

The interview will be semi-structured, and we suggest using a conversational style. There is no need to follow the suggested topics in this order or to ask every prompting question, the important thing is to ensure each topic is discussed at some point during the interview, and this may mean that it is brought up by the participant without needing to be prompted. It may be useful to cross out rows as the participant addresses them so that you don't ask them to repeat information they have already shared. After the interviewer introduces themselves, we suggest adding:

"We're going to speak to you today about your own experiences as an ASHA, particularly how you have worked with your ASHA Sangini since they started using a phone app to supervise and support you. CommCare app was rolled out. Through this project, we are hoping to learn how the app might have changed the work of ASHAs and ASHA Sanginis. I have a few different topics I would like to cover, but please feel welcome to share your thoughts as they come to you, even if I haven't asked a specific question on that topic.

Knowing about the purpose of this project and having looked at the findings of the previous project, is there anything you'd like to share with me to begin with?"

**Topic**	**Argument for inclusion / guidance for interviewer**	**Potential prompts**<br>**(remember, you DO NOT have to ask all of these prompts – they are there in case you need more ideas for how to broach topics)**
Introduction		
Demographic information	It is useful to know a few basic facts about the ASHA, so that we can compare between interviews. Please find out:<br>• Their location (and whether it has changed)<br>• Their age<br>• Their level of education and literacy/numeracy	It is useful for us to know a bit about you so that we can understand your experiences better.<br>• How long have you been in *location*? Where did you live before that?<br>• Can you please tell me your age?<br>• Can you please tell me the highest level of school that you attended?
On being an ASHA		
Core details related to being an ASHA	We would like to know (please ensure all of these are discussed):<br>• How long the ASHA has been in their role, particularly to understand their experiences before and after the introduction of the app<br>• Who their ASHA Sangini is, so that we can compare their perceptions of supervision<br>• How often they meet with their ASHA Sangini and how long for	When did you first become an ASHA?<br>Have you been an ASHA since then, or have you taken breaks?<br>Who is your current ASHA Sangini? How long have they been your ASHA Sangini?<br>Have you had other ASHA Sanginis in the past?<br>How often do you have a supervision visit with your ASHA Sangini?<br>How long do supervision meetings tend to be?

Topic	Argument for inclusion / guidance for interviewer	Potential prompts<br><br>(remember, you DO NOT have to ask all of these prompts – they are there in case you need more ideas for how to broach topics)
Experiences as an ASHA	We would like to understand the role of each ASHA, and how it may differ to other ASHAs (please ensure all of these are discussed):<br>• How often they visit pregnant women<br>• How often they visit individual pregnant women<br>o Does this change if it is a high-risk pregnancy?<br>• How long visits tend to be<br>• How long they spend recording notes in their diary	Can you tell me a bit more about your work? What does it entail?<br><br>How often do you visit pregnant women?<br><br>How often do you visit each individual pregnant woman? Does this depend on anything?<br><br>How long do you normally visit with pregnant women for?<br><br>How long do you spend recording notes in your diary? Do you do this during the visit, or afterwards?
Supervision visits	It is useful to know what a typical supervision meeting entails, so that we can understand if ASHA Sanginis conduct their meetings differently. We are interested in (please ensure all of these are discussed):<br>• What happens at the start of a supervision meeting<br>• Whether ASHA Sanignis look at the ASHA's diary<br>• Whether ASHA Sanginis visit a selection of pregnant women listed in the ASHA's diary (and if so, what happens during these visits)<br>• Whether the ASHA Sangini tracks what is in their drug kit (only relevant for ASHAs in the five districts, not the state-wide scale up)<br>• The kinds of feedback typically provided to them by their ASHA Sangini<br><br>The interviewee may provide all of these points with one prompt (e.g., "what happens during a typical supervision meeting?" or might require more specific prompts (e.g., "does your ASHA Sanigini look at your diary"?). Please use your judgement.	I understand that you have regular meetings with your ASHA Sangini – can you tell me what typically happens during these?<br><br>What happens at the beginning of a supervision meeting?<br><br>Does your ASHA Sangini look at your written diary?<br><br>Does your ASHA Sangini visit some of the women listed in your diary? Are there certain types of women they tend to visit? (e.g., women with high risk pregnancies, but do not provide suggestions as this can bias responses)<br><br>Does your ASHA Sangini track what is in your drug kit?<br><br>What kind of feedback do you normally receive from your ASHA Sangini?
Changes in supervision practice	It is useful to know whether their supervisory practice has changed over time, and it is something we can compare across multiple interviews. The participant might start talking about the app here, but it's ok if they don't.	Have your supervision meetings changed over time?<br><br>Has this change been because you had a new ASHA Sangini or something else?<br><br>Are there things your ASHA Sangini does now that they didn't do earlier?

Topic	Argument for inclusion / guidance for interviewer	Potential prompts<br>(remember, you DO NOT have to ask all of these prompts – they are there in case you need more ideas for how to broach topics)
Use of the app		
Perceptions of the app	We would like to know whether the ASHA has noticed any changes to their supervision specifically related to the introduction of the app, or if there is anything they would like to share in general about their ASHA Sanigni's use of the app	Has anything changed during your supervision meetings since the app was introduced?<br>• Before the app, how did your ASHA Sangini keep track of your work?<br>• Before the app, how did your ASHA Sangini keep track of medical events?<br>Has your role become easier/harder since the app was introduced? Can you please share any significant changes?<br>Is there anything you'd like to share generally about the app and what happens in your supervision meetings?
Specific app functions	ASHAs may not be able to comment on specific features of the app, but we would like to ask them about two functions in case they have useful insights to share:<br>• The expected beneficiary form<br>• The indicators of functionality form<br>• The drug kit tracking form (only relevant for ASHAs in the five districts, not the state-wide scale up)<br>• Reporting/tracking health events (e.g., high risk pregnancies, maternal deaths, child deaths)<br>They may have discussed these already – if they have not, please prompt them to speak about each task.	Is there anything you would like to share about how your ASHA Sangini uses the app to fill in the expected beneficiary form and communicate details to you?<br>Is there anything you would like to share about how your ASHA Sangini uses the app to record your functionality?<br>Is there anything you would like to share about how your ASHA Sangini uses the app to track your drug kit?<br>Is there anything you would like to share about how your ASHA Sangini uses the app to record health events (e.g., high risk pregnancies, maternal deaths, child deaths)??
Experiences generally: We have asked participants to give up their valuable time to take part in this research. They may appreciate the opportunity to have their voices heard on other aspects of their role, besides the app, and we should allow and encourage this		
Experiences generally	(if they haven't been forthcoming earlier in interview and more prompting would be useful)	Can you tell us about your experiences more broadly as an ASHA?<br>What is good/bad, hard/easy, enjoyable/challenging about your role?<br>Is there anything you would like to share about your experiences being supervised by ASHA Sanginis?
Close of interview		
	It is best practice to ask whether the participant has anything more they would like to share before closing the interview and thanking them	Is there anything else you'd like to share with me about your experience as an ASHA?
